# 3-(4-Hydroxy-3-methoxyphenyl)propionic Acid Produced from 4-Hydroxy-3-methoxycinnamic Acid by Gut Microbiota Improves Host Metabolic Condition in Diet-Induced Obese Mice

**DOI:** 10.3390/nu11051036

**Published:** 2019-05-09

**Authors:** Ryuji Ohue-Kitano, Satsuki Taira, Keita Watanabe, Yuki Masujima, Toru Kuboshima, Junki Miyamoto, Yosuke Nishitani, Hideaki Kawakami, Hiroshige Kuwahara, Ikuo Kimura

**Affiliations:** 1 Department of Applied Biological Science, Graduate School of Agriculture, Tokyo University of Agriculture and Technology, Fuchu-shi, Tokyo 183-8509, Japan; s183332q@st.go.tuat.ac.jp (S.T.); s177090s@st.go.tuat.ac.jp (K.W.); fu3265@go.tuat.ac.jp (Y.M.); s170292x@st.go.tuat.ac.jp (T.K.); m-junki@go.tuat.ac.jp (J.M.); 2 Research Center, Maruzen Pharmaceuticals Co., Ltd., Fukuyama, Hiroshima 729-3102, Japan; y-nishitani@maruzenpcy.co.jp (Y.N.); h-kawakami@maruzenpcy.co.jp (H.K.); h-kuwahara@maruzenpcy.co.jp (H.K.)

**Keywords:** 4-hydroxy-3-methoxycinnamic acid, 3-(4-hydroxy-3-methoxyphenyl)propionic acid, high-fat diet, obesity, hepatic lipid metabolism, gut microbiota

## Abstract

4-Hydroxy-3-methoxycinnamic acid (HMCA), a hydroxycinnamic acid derivative, is abundant in fruits and vegetables, including oranges, carrots, rice bran, and coffee beans. Several beneficial effects of HMCA have been reported, including improvement of metabolic abnormalities in animal models and human studies. However, its mitigating effects on high-fat diet (HFD)-induced obesity, and the mechanism underlying these effects, remain to be elucidated. In this study, we demonstrated that dietary HMCA was efficacious against HFD-induced weight gain and hepatic steatosis, and that it improved insulin sensitivity. These metabolic benefits of HMCA were ascribable to 3-(4-hydroxy-3-methoxyphenyl)propionic acid (HMPA) produced by gut microbiota. Moreover, conversion of HMCA into HMPA was attributable to a wide variety of microbes belonging to the phylum Bacteroidetes. We further showed that HMPA modulated gut microbes associated with host metabolic homeostasis by increasing the abundance of organisms belonging to the phylum Bacteroidetes and reducing the abundance of the phylum Firmicutes. Collectively, these results suggest that HMPA derived from HMCA is metabolically beneficial, and regulates hepatic lipid metabolism, insulin sensitivity, and the gut microbial community. Our results provide insights for the development of functional foods and preventive medicines, based on the microbiota of the intestinal environment, for the prevention of metabolic disorders.

## 1. Introduction

Dietary phenolic compounds are secondary metabolites in plants, and they are valued for their health benefits. Among foods containing phenolic phytochemicals, coffee is one of the most widely consumed beverages, and it is an extremely rich source of chlorogenic acid (CGA) and caffeic acid (CA) [1]. Indeed, several studies have indicated that habitual coffee consumption potentially has several beneficial health effects, including the prevention of heart disease and stroke, cancer risk reduction, and improvement of metabolic disease [2].

4-Hydroxy-3-methoxycinnamic acid (HMCA), which is biosynthesized from CGA and CA by O-methyltransferase (3-O-methyltransferase), is an abundant hydroxycinnamic acid-derived metabolite in plants [3,4]. HMCA is reported to have antioxidant and anti-inflammatory effects, as well as anticancer properties. It is also thought to improve cognition and neurodegeneration, and to play a role in the regulation of bone remodeling [5,6,7,8,9]. Additionally, recent investigations demonstrated that HMCA exerts beneficial metabolic effects, including abrogation of glucose dysregulation, dyslipidemia, and inflammation in both animal models and humans [10,11]. However, little is known about the molecular mechanisms that regulate metabolic syndrome and associated diseases as a result of dietary HMCA intake.

The gut microbiota affects host metabolic homeostasis through the fermentation of a wide range of indigestible compounds of plant origin [12,13]. A recent clinical study demonstrated that CGA derivatives, along with CA and HMCA, are absorbed in the small intestine, whereas other metabolites, such as dihydrocaffeic acid and 3-(4-hydroxy-3-methoxyphenyl)propionic acid (HMPA), are produced in the large intestine [14]. Thus, these reports suggest that the gut microbiota is mainly responsible for the formation and conversion of CGA-derived metabolites in the intestine, and that these metabolites may play a role in host homeostasis. Furthermore, several studies have demonstrated that HMPA is associated with intestinal anti-inflammation, and that it has antioxidant and neuroprotective activities [15,16]. However, few studies have investigated the effects of HMPA on metabolic control.

In this study, we investigated the beneficial metabolic effects of HMCA in a mouse model of high-fat diet (HFD)-induced obesity, as well as the molecular mechanism underlying these effects. Furthermore, we examined whether gut bacteria produced HMPA following intake of HMCA in the intestine, and whether dietary HMPA intake exerts beneficial metabolic effects similar to those resulting from HMCA consumption. Finally, we evaluated the effects of HMCA and HMPA on the gut microbial community. 

## 2. Materials and Methods

### 2.1. Mice, Diet, and Experimental Design

Male C57BL/6J mice were purchased from Japan SLC (Shizuoka, Japan) and maintained under a strict 12 h light/dark cycle and were housed in a conventional animal room at 23.0 °C. Mice were acclimated to the laboratory conditions on the CLEA Rodent Diet (CE-2, CLEA Japan, Inc., Tokyo, Japan) for 1 week prior to the treatment. The 4-week-old C57BL/6J mice were placed on a D12492 diet (HFD; 60% kcal fat, Research diets, New Brunswick, NJ, USA) or HFD containing 1% HMCA for 12 weeks (*n* = 7–9). The compositions of the diets are given in Table 1. In another experiment, the 4-week-old mice were divided into two groups of similar average body weight (groups fed HFD supplemented with 1% cellulose or 1% HMPA) for 12 weeks in a factorial design (*n* = 7–9). The compositions of the diets are given in Table 2. These diets were adjusted so that the final percentages of protein, fat, and carbohydrates were almost equal. The control group diet was supplemented with 1% cellulose in the HFD. HMCA and HMPA were supplied by Maruzen Pharmaceuticals Co., Ltd. (Hiroshima, Japan) During the treatment, body weights were measured once a week. Food intake was measured every 2 or 3 days for 12 weeks, and the average of the daily food intake (g/day/mouse) was calculated (Tables S1 and S2). For the antibiotic treatment, 4-week-old mice were treated with ampicillin (Nacalai Tesque, Kyoto, Japan; 0.4 mg/mL), neomycin (Nacalai Tesque; 0.4 mg/mL), metronidazole (Wako, Tokyo, Japan; 0.4 mg/mL), gentamicin (Sigma-Aldrich, St. Louis, MO, USA; 0.4 mg/mL), and vancomycin (Sigma-Aldrich; 0.2 mg/mL) in drinking water for 2 weeks. Mice treated with/without antibiotics were fed HFD containing HMCA for one week. After feeding, the cecal contents of HMCA and HMPA were determined. All mice were then sacrificed under deep isoflurane-induced anesthesia. Liver, cecum, epididymal, perirenal, and subcutaneous adipose tissues, and brown adipose tissue (BAT) were harvested and weighted. Blood was collected from the inferior vena cava using heparinized tubes and plasma was separated by immediate centrifugation (7000× *g*, 5 min, 4 °C). All tissues and plasma were stored at −80 °C until further processing. All experimental procedures involving mice were planned in accordance with the guidelines of the Committee on the Ethics of Animal Experiments of the Tokyo University of Agriculture and Technology (permit number: 28–87). All efforts were made to minimize suffering.

### 2.2. Plasma Biochemical Analyses

Blood glucose was assessed using a portable glucometer with compatible glucose test strips (OneTouch^®^Ultra^®^, LifeScan, Milpitas, CA, USA). Plasma cholesterol (LabAssay™ Cholesterol, Wako, Tokyo, Japan), non-esterified fatty acids (NEFAs) (LabAssay™ NEFA, Wako, Tokyo, Japan), triglyceride (TG) (LabAssay™ Triglyceride, Wako, Tokyo, Japan), and insulin (Insulin ELISA KIT (RTU), Shibayagi, Gunma, Japan) were measured using commercial assay kits following manufacturer’s instructions. 

### 2.3. Quantification of Hepatic Triglyceride Content

Livers were weighed, and stored at −80 °C. Hepatic triacylglycerol contents were measured following a modified protocol as previously described [17]. 

### 2.4. Hepatic Histology

Liver were embedded in OCT compound (Sakura Finetek Japan, Tokyo, Japan) and sectioned at 8 μm. These cryosections were stained with hematoxylin and eosin (H&E).

### 2.5. Quantification of Phytochemicals by HPLC

Mouse plasma and tissue samples were dissolved or homogenized in extraction solvent (acetonitrile). HMCA and HMPA in cecal and plasma were analyzed using an Agilent 1200 HPLC with a DAD detector (at 320 nm for HMCA and 280 nm for HMPA) (Agilent Technologies, Santa Clara, CA, USA). Separations were performed at 40 °C using a Wakosil-II 5C18 H (4.6 mm × 150 mm) (Wako, Tokyo, Japan). Injections were carried out with an autosampler maintained at 4 °C. The mobile phase was pumped at a flow rate of 1 mL/min. 

### 2.6. Analysis of Gut Microbiota by 16S rRNA Gene Sequencing

Cecal DNA was extracted using FastDNA^®^ SPIN Kit for Feces (MP Biomedicals, Santa Ana, CA, USA). The V4 region of the 16S rRNA gene was amplified using dual-indexed primers. The amplicons were sequenced using an Illumina MiSeq with a MiSeq Reagent kit V3 (Illumina, San Diego, CA, USA). Paired-end sequencing was carried out using Illumina MiSeq platform. Processing and quality filtering of reads were performed with Quantitative Insights into Microbial Ecology (QIIME) (v1.9.1) and the chimera-free sequences were aligned with the SILVA database (http://www.arb-silva.de) at 97% identity. The raw data have been deposited into the DNA Data Bank of Japan (DDBJ) database under accession no. DRA008102 and DRA008103.

### 2.7. Real Time-PCR (RT-PCR)

RT-PCR protocol was conducted following a modified protocol as previously described [17]. Reverse transcription were performed using Moloney murine leukemia virus reverse transcriptase (Invitrogen, Carlsbad, CA, USA). Real-time PCR was completed with using SYBR Premix Ex Taq II (TaKaRa, Shiga, JAPAN) and the StepOne^TM^ real time PCR system (Applied Biosystems, Foster City, CA, USA). The ΔΔct method for PCR and *18S* typically was used as the housekeeping mRNA. Primer sequences are shown in Table 3. 

### 2.8. Statistical Analysis

Statistical comparisons between diet groups were performed with using a two-tailed paired Student’s *t*-test and one-way ANOVA followed by Dunnett’s multiple comparison test. The false discovery rate (FDR) *q*-value in the 16S rDNA sequencing was analyzed. The FDR (*q*-value) was estimated with the Benjaminie–Hochberg procedure. 16S rDNA sequencing data were analyzed by Student’s *t*-test with the FDR correction. *p*-values < 0.05 and *q*-value < 0.1 were considered significant. Data analysis was performed using GraphPad Prism 7.0 (Graphpad Software, San Diego, CA, USA), and data are presented as means ± SEM. 

## 3. Results

### 3.1. HMCA Intake Suppresses HFD-Induced Obesity

**Figure 1:**
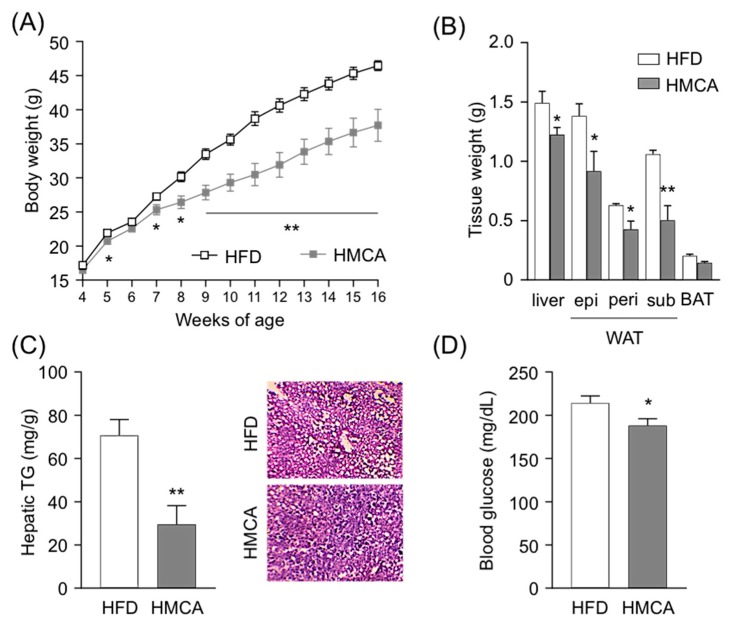
Metabolic parameters and histological changes in high-fat diet (HFD)- and 4-hydroxy-3-methoxycinnamic acid (HMCA)-supplemented HFD-fed mice. Mice were characterized for body weight gain (**A**), the mass of WAT, BAT, and liver (**B**), hepatic TG and histology of hepatocytes by hematoxylin and eosin (H&E) staining (**C**), and blood glucose (**D**) (*n* = 7–8). All data are presented as the means ± SEM. Differences were assessed by Student’s *t*-test. Significance is established at adjusted ** *p* < 0.01 and * *p* < 0.05. WAT: white adipose tissue; BAT: brown adipose tissue; TG: triglyceride.

We first investigated changes in metabolic parameters following HMCA feeding in a mouse model of HFD-induced obesity. In this experiment, 4-week-old mice were fed an HFD containing 1% HMCA for 12 weeks (Table 1), and tissue characteristics and biochemical parameters were assessed at the end of the treatment. Gains in body weight were significantly lower in HMCA-supplemented HFD-fed mice during growth (Figure 1A). The mass of white adipose tissue (WAT) was also significantly lower in HMCA-supplemented HFD-fed mice than in those fed an HFD, whereas no significant difference was observed in the weight of BAT (Figure 1B). Moreover, a significant decrease in the weight of livers from HMCA-supplemented HFD-fed mice was observed, and corresponded to a decrease in hepatic TG in comparison to HFD-fed mice (Figure 1B,C). Plasma levels of glucose, insulin, and total cholesterol were significantly lower in HMCA-supplemented HFD-fed mice (Figure 1D, Figure S1A,B), whereas their TG and NEFAs plasma levels were comparable (Figure S1B). HMCA intake suppressed both elevation of plasma glucose levels and increases in hepatic TG accumulation, thereby preventing HFD-induced obesity.

### 3.2. Gut Microbiota Convert HMCA into HMPA in the Intestine

**Figure 2:**
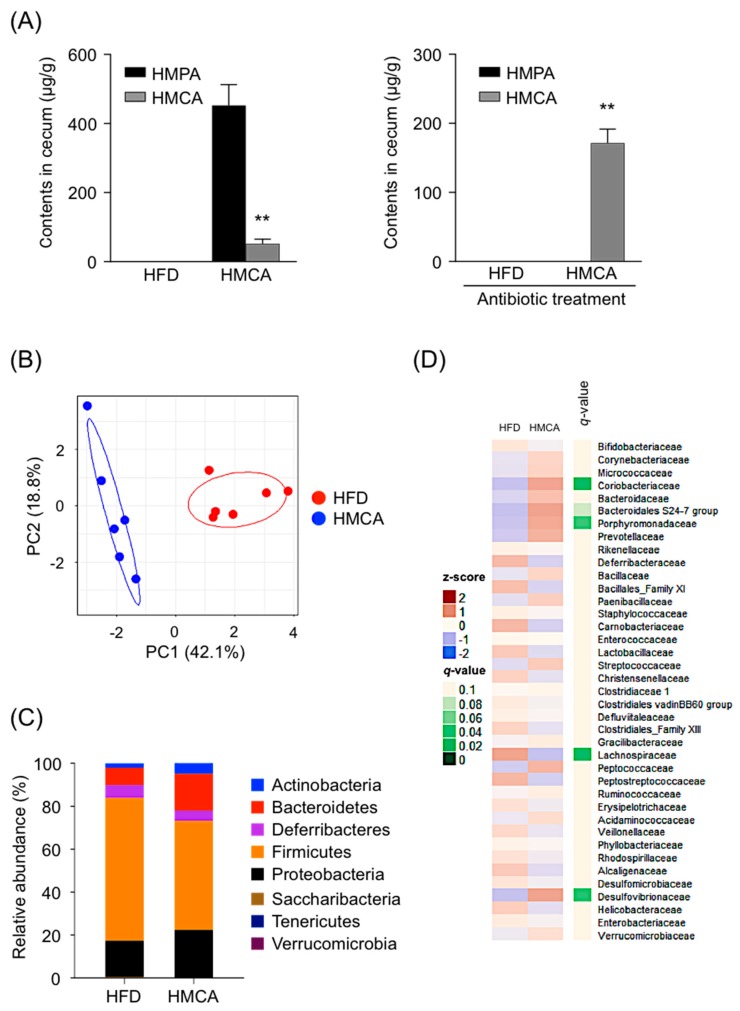
Conversion of HMCA into HMPA by gut microbiota. (**A**) Mice treated with/without antibiotics were fed HFD containing HMCA for one week. After feeding, the cecal contents of HMCA and HMPA were determined (*n* = 6–8, left; conventional condition, right; antibiotic treatment). (**B**–**D**) Changes in the gut microbiome in the HMCA-supplemented HFD-fed mice for 12 weeks. (B) Principal coordinate analysis (PCoA) of the cecal microbiota in HMCA-supplemented HFD-fed vs. the HFD-fed mice based on unweighted Unifrac distances between diet treatments (*n* = 6). (**C**) Relative abundance of the phylum level (*n* = 6). (**D**) Heatmap of relative abundance of major taxonomic groups at family level (mean relative abundance >0.1%) in HMCA-supplemented HFD-fed mice vs. the HFD-fed control mice (*n* = 6). FDR, *q* < 0.1. All data are presented as the means ± SEM. Differences were assessed by Student’s *t*-test. Significance is established at adjusted ** *p* < 0.01. HFD: high-fat diet; HMCA: 4-hydroxy-3-methoxycinnamic acid; HMPA: 3-(4-hydroxy-3-methoxyphenyl)propionic acid.

We identified and quantified HMCA and HMPA contents in cecal samples collected following 1 week of HFD containing with 1% HMCA in the mice. Interestingly, HMPA but not HMCA was detected in the cecum of HMCA-supplemented HFD-fed mice (Figure 2A; left), whereas only HMCA was detected when mice received antibiotic treatment (Figure 2A; right). Then, HMPA was detected in the urine of mice fed HFD supplemented with HMCA or HMPA (Figure S2A). In addition, the plasma pharmacokinetic profiles following intraperitoneal injection showed that there was no possibility of interconversion between HMCA and HMPA by the host metabolism (Figure S2B,C). These data support the idea that HMCA taken orally could be converted into HMPA in the intestine by gut microbiota and that HMPA was absorbed in the body. We evaluated the composition of gut microbiota in HFD-fed and HMCA-supplemented HFD-fed mice. Principal coordinate analysis (PCoA) based on unweighted Unifrac distances indicated significant clustering by diet type, with complete separation of the cecal microbiota of HMCA-supplemented HFD-fed mice from that of HFD controls along the PCoA1 axis (Figure 2B). Taxonomic analysis of the cecal microbiota showed an increased abundance of Bacteroidetes and Actinobacteria, with a reduction in the population of Firmicutes in the HFD-HMCA microbiota (Figure 2C). Furthermore, the hierarchical clustering of individual families also confirmed that HMCA intervention resulted in an increase in the population of the Bacteroidales S24-7 group and Porphyromonadaceae, which belong to the phylum Bacteroidetes (Figure 2D).

### 3.3. HMPA is Molecular Entity Underlying Metabolic Improvement Following HMCA Intake

**Figure 3:**
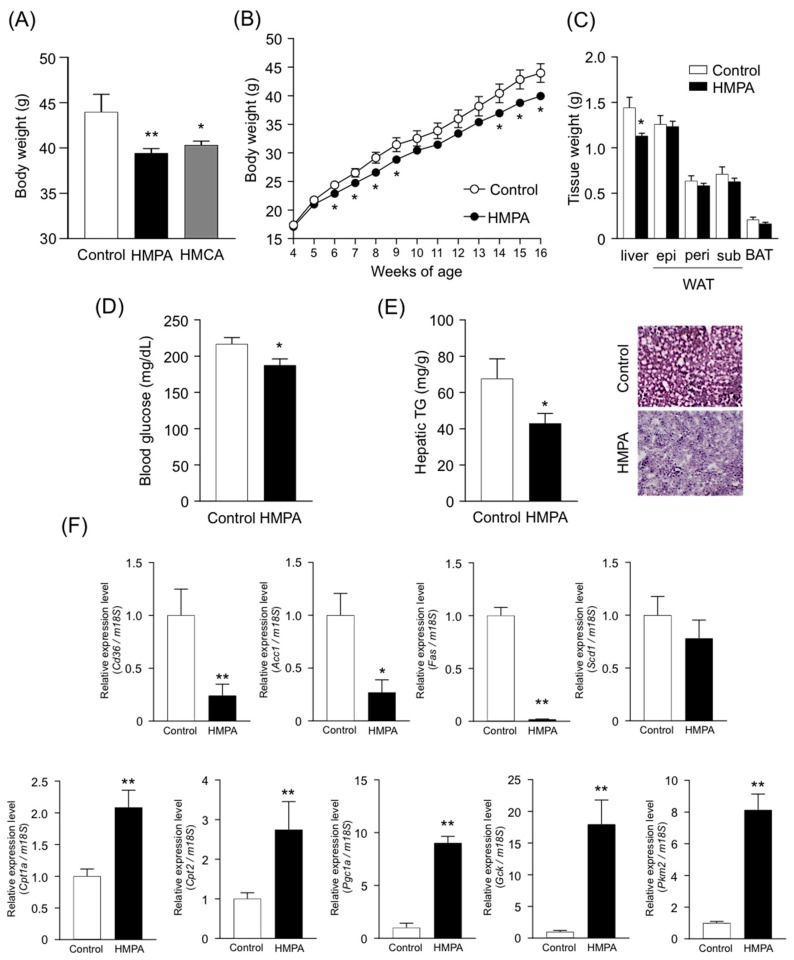
Metabolic parameters and histological changes in HMPA-supplemented HFD-fed mice. (**A**) Body weights after feeding control diet, HFD containing HMCA, and HFD containing HMPA for 12 weeks. (**B**–**E**) Metabolic parameters and histological changes in HFD controls and HMPA-supplemented HFD-fed mice. Mice were characterized for body weight gain (**B**), the mass of WAT, BAT, and liver (**C**), blood glucose (**D**), and hepatic TG and histology of hepatocytes by H&E staining (**E**) (*n* = 7–9). (**F**) Relative mRNA expressions involved in energy expenditure (*Pgc1a*), β-oxidation (*Cpt1a* and *Cpt2*), fatty acid synthesis (*Fas*, *Acc1*, and *Scd1*), fatty acid trafficking (*Cd36*), and glycolysis (*Gck* and *Pkm2*) in the liver of HFD controls and HMPA-supplemented HFD-fed mice (*n* = 7). Results are expressed as fold mRNA change vs. HFD control; values were set to 1. To compare mRNA expression levels among samples, copy numbers of all transcripts were normalized to *18S* mRNA expression levels, an internal control. All data are presented as the means ± SEM. Differences were assessed using one-way ANOVA followed by Dunnett’s multiple comparison test (**A**) and by Student’s *t*-test (**B**–**F**). Significance is established at adjusted ** *p* < 0.01, and * *p* < 0.05. Control, HFD containing with 1% cellulose fed mice. HFD: high-fat diet; HMCA: 4-hydroxy-3-methoxycinnamic acid; HMPA: 3-(4-hydroxy-3-methoxyphenyl)propionic acid.

As shown in Figure 2, HMPA is produced by gut microbiota in the intestine following HMCA intake. We therefore examined whether dietary HMPA intake directly affects metabolic parameters in a mouse model of HFD-induced obesity (Table 2). After feeding for 12 weeks, the body weights of HMPA-supplemented HFD-fed mice were comparable to those of HMCA-supplemented HFD-fed mice, and significantly lower than those of mice fed a control diet (Figure 3A). Additionally, gains in body weights were significantly lower in HMPA-supplemented HFD-fed mice during growth (Figure 3B). Liver weights were also significantly lower in HMPA-supplemented HFD-fed mice compared to the controls (Figure 3C). Moreover, significant decreases in plasma glucose levels were observed in HMPA-supplemented HFD-fed mice and were accompanied by decreased plasma insulin levels (Figure 3D, Figure S3A). In contrast, plasma levels of total cholesterol, TG, and NEFAs were similar between controls and HMPA-supplemented HFD-fed mice (Figure S3B). HMPA intake led to a significant reduction in hepatic TG accumulation, and the H&E-stained hepatic sections obtained from HMPA-supplemented HFD-fed mice showed reduced cytoplasmic vacuolation. This indicated an alleviation in steatosis in comparison with the controls (Figure 3E). HMPA intake therefore exerts beneficial metabolic effects to a similar extent as dietary HMCA intake. We also investigated the expression profiles of hepatic genes related to energy metabolism. The expression of genes related to energy expenditure, glycolysis, and β-oxidation increased, while that of genes related to fatty acid synthesis and trafficking were decreased in HMPA-supplemented HFD-fed mice compared to controls (Figure 3F). 

### 3.4. HMPA Intake Modifies the Changes of HFD-Associated Gut Microbial Composition

**Figure 4:**
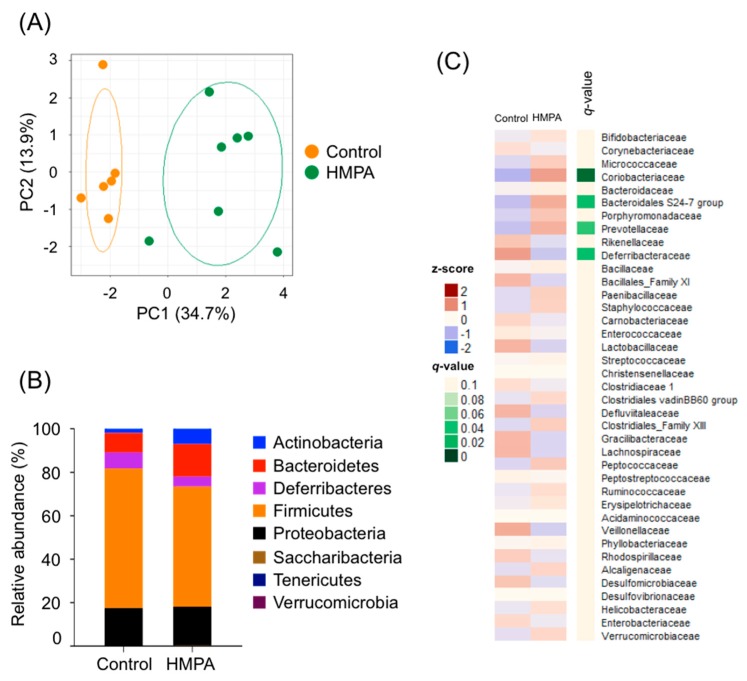
Compositions of gut microbiota in the HMPA-supplemented HFD-fed mice compared to the HFD controls. (**A**) PCoA of the cecal microbiota in HMPA-supplemented HFD-fed mice vs. the HFD controls based on unweighted Unifrac distances between diet groups (*n* = 6–7). (**B**) Relative abundance of the phylum level (*n* = 6–7). (**C**) Heatmap of relative abundance of major taxonomic groups at family level (mean relative abundance >0.1%) in HMPA-supplemented HFD-fed mice vs. the HFD controls (*n* = 6–7). FDR, *q* < 0.1. All data are presented as the means ± SEM. Differences were assessed by Student’s *t*-test. Significance is established at adjusted *p* < 0.05. Control, HFD containing with 1% cellulose fed mice. HFD: high-fat diet; HMPA: 3-(4-hydroxy-3-methoxyphenyl)propionic acid.

We further investigated changes in the composition of gut microbiota following HMPA consumption. PCoA based on unweighted Unifrac distances indicated significant clustering by diet type, with complete separation of the cecal microbiota of HMPA-supplemented HFD-fed mice from that of the HFD-fed controls (Figure 4A). Taxonomic analysis of the cecal microbiota showed an increased abundance of Bacteroidetes, along with a reduction in the population of Firmicutes with HMPA supplementation (Figure 4B). Notably, HMPA intervention induced a drastic increase in the population of Actinobacteria, while the population of Deferribacteres in the cecal microbiota of HMPA-supplemented HFD-fed mice were decreased (Figure 4B). These results were similar to those observed with HMCA supplementation. Hierarchical clustering of individual families also confirmed the effect of HMPA intake on gut microbiota (Figure 4C). The HMPA intervention significantly increased the Bacteroidales S24-7 group and Coriobacteriaceae, which are families within the Bacteroidetes and Actinobacteria, respectively (Figure 4C). These families were also significantly increased in HMCA-supplemented HFD-fed mice (Figure 2D). Meanwhile, significant increases in Prevotellaceae and decreases in Deferribacteraceae were observed in HMPA-supplemented HFD-fed mice, but not in HMCA-supplemented HFD-fed mice (Figure 2D and Figure 4C). These findings demonstrated that HMPA intake changed the composition of mouse gut microbiota.

## 4. Discussion

There is growing evidence that the intestinal tract plays an important role in the catabolism and bioavailability of dietary phenolic compounds, and that their metabolites can have an impact on both host metabolic homeostasis and the diversity of the gut microbiome. Our work in mice, using an HFD containing HMCA, confirmed earlier results that support the metabolic benefits of HMCA, including abrogation of hepatic lipid accumulation and improvement of HFD-induced insulin resistance [10,11,18]. However, the molecular mechanisms underlying this effect remained unclear in previous studies. Herein, we showed that microbiota-derived HMPA significantly contributed to beneficial metabolic effects in obese HFD-induced mice. HMPA directly improved hepatic lipid metabolism and insulin sensitivity. Indeed, several studies have shown that HMPA is efficiently transported together with HMCA in the intestine [19,20]; hence, HMPA would substantially contribute to improved human health. However, HMCA absorbed from the intestine is converted into various derivatives other than HMPA in the liver [20]. Therefore, further experiments would be required to determine the nature of the indirect biological effects of other HMCA-derived metabolites.

A previous report highlighted the potential of dietary phytochemicals to modulate the gut microbiota in a beneficial way [21]. Our results demonstrated that the families Bacteroidales S24-7 and Coriobacteriaceae were significantly increased in both HMPA-fed and HMCA-fed mice. Furthermore, a recent study revealed that Actinobacteria of the family Coriobacteriaceae might also be involved in the stimulation of hepatic detoxification activity, with the strongest effect being on hepatic lipid metabolism [22]. This evidence suggests that these Coriobacteriaceae are associated with improved hepatic metabolism following HMCA and/or HMPA consumption.

Recent investigations have demonstrated that lactic acid bacteria are highly resistant to antimicrobial phenolic acids, and that this resistance is partially dependent on their capacity to convert phenolic acids to metabolites with lower metabolic activities [23]. Indeed, several plant-associated bacteria, such as *Lactobacillus curvatus* (*L*. *curvatus*), *L. plantarum*, *L. rossiae*, *Weissella cibaria* (W. *cibaria*), and *W. confusa*, reduce HMCA as external acceptors of electrons, and consequently produce HMPA [24,25]. Additionally, Santamaría et al. revealed that *L. plantarum* possesses hydroxycinnamate reductase, which is a heterodimeric NADH-dependent coumarate reductase that catalyzes the conversion of HMCA to HMPA [26]. However, we found that HMCA led to a decrease in the abundance of certain lactic acid bacteria (e.g., species from the Lactobacillaceae and Lachnospiraceae), whereas a wide variety of microbes belonging to the phylum Bacteroidetes was increased. Interestingly, we observed that the population of organisms belonging to Bacteroidaceae and Porphyromonadaceae were increased with HMCA supplementation, but not with HMPA supplementation, suggesting that conversion of HMCA into HMPA in the intestine is carried out by members of the Bacteroidetes. 

In this study, we demonstrated that dietary HMCA was efficacious against diet-induced weight gain and hepatic steatosis, modulating the community structure of gut microbes. We further showed that dietary HMCA exerts beneficial metabolic effects via the modulation of hepatic lipid metabolism. These effects occur as a result of HMPA production by the gut microbiota, and through increases in beneficial gut microbes, suggesting that HMPA is a molecular entity underlying metabolic improvement observed with HMCA consumption. Functional alterations to the gut microbiome in response to HMPA consumption warrant detailed follow-up investigations, and further characterization at the species level of the specific gut microbes involved in the reduction of HMCA. Our results may contribute to the development of functional foods for the prevention of metabolic disorders, including obesity and type 2 diabetes. The study also provides information that could facilitate the development of preventive medicines based on the microbiota of the intestinal environment. 

## 5. Conclusions

This study describes the mitigating effects of 4-hydroxy-3-methoxycinnamic acid (HMCA) on high fat diet-induced obesity using a mouse model and shows that these effects are due to 3-(4-hydroxy-3-methoxyphenyl)propionic acid (HMPA) produced from HMCA by the gut microflora. It also demonstrates gut microbiota-modulating effects of HMPA. We believe that our study makes a significant contribution to the literature because it demonstrates that the beneficial effects of dietary HMCA are due to the modulation of hepatic lipid metabolism.

## Figures and Tables

**Table 1:** Diet compositions.

Formula	HFD ^1^	HMCA ^2^
Product	kcal %	kcal %
Protein	20	20
Carbohydrate	20	20
Fat	60	60
Ingredient	gm	gm
Casein, 30 mesh	200	198
L-cystine	3	2.97
Corn starch	0	0
Maltodextrin 10	125	123.75
Sucrose	68.8	68.112
Cellulose, BW200	50	49.5
HMCA	0	7.7385
Cellulose	0	0
Soybean oil	25	24.75
Lard	245	242.55
Mineral mix S10026	10	9.9
Dicalcium phosphate	13	12.87
Calcium carbonate	5.5	5.445
Potassium citrate, 1 H_2_O	16.5	16.335
Vitamin mix V10001	10	9.9
Choline bitartrate	2	1.98
FD&C blue dye #1	0.05	0.0495

^1^ HFD: high-fat diet; ^2^ HMCA: HFD + 1% 4-hydroxy-3-methoxycinnamic acid. FD&C blue dye #1: synthetic organic compound primarily used as a blue colorant for dietary supplements.

**Table 2:** Diet compositions.

Formula	Control ^1^	HMPA ^2^
Product	kcal %	kcal %
Protein	20	20
Carbohydrate	20	20
Fat	60	60
Ingredient	gm	gm
Casein, 30 mesh	198	198
L-cystine	2.97	2.97
Corn starch	0	0
Maltodextrin 10	123.75	123.75
Sucrose	68.112	68.112
Cellulose, BW200	49.5	49.5
HMPA	0	7.7385
Cellulose	7.7385	0
Soybean oil	24.75	24.75
Lard	242.55	242.55
Mineral mix S10026	9.9	9.9
Dicalcium phosphate	12.87	12.87
Calcium carbonate	5.445	5.445
Potassium citrate, 1 H_2_O	16.335	16.335
Vitamin mix V10001	9.9	9.9
Choline bitartrate	1.98	1.98
FD&C blue dye #1	0.0495	0.0495

^1^ Control: HFD + 1% cellulose; ^2^ HMPA: HFD + 1% 3-(4-hydroxy-3-methoxyphenyl)propionic acid. FD&C blue dye #1: synthetic organic compound primarily used as a blue colorant for dietary supplements.

**Table 3:** Primer sequences used in this study.

Gene	Primer	Sequence
*18S*	Forward	5′-CTCAACACGGGAAACCTCAC-3′
	Reverse	5′-AGACAAATCGCTCCACCAAC-3′
*Pgc1a*	Forward	5′-GAGAATGAGGCAAACTTGCTAGCG-3′
	Reverse	5′-TGCATGGTTCTGAGTGCTAAGACC-3′
*Acc1*	Forward	5′-AAGGCTATGTGAAGGATG-3′
	Reverse	5′-CTGTCTGAAGAGGTTAGG-3′
*Cpt1a*	Forward	5′-GCATAAACGCAGAGCATTCC-3′
	Reverse	5′-GATGTTGGGGTTCTTGTCTCC-3′
*Cpt2*	Forward	5′-CTCATCCGCTTTGTTCCTTC-3′
	Reverse	5′-AGTTCATCACGACTGGGTTTG-3′
*Cd36*	Forward	5′-TGGCAAAGAACAGCAGCAAA-3′
	Reverse	5′-GACAGTGAAGGCTCAAAGATGG-3′
*Pkm2*	Forward	5′-TCTTCTGGACCCATCGGCCCCAGGA-3′
	Reverse	5′-AAAGGGATAGGGGAGGGGAAG-3′
*Fas*	Forward	5′-GCTGCGGAAACTTCAGGAAAT-3′
	Reverse	5′-AGAGACGTGTCACTCCTGGACTT-3′
*Scd1*	Forward	5′-GTCAGGAGGGCAGGTTTC-3′
	Reverse	5′-GAGCGTGGACTTCGGTTC-3′
*Gck*	Forward	5′-TACCCCTGGGCTTCACCTT-3′
	Reverse	5′-CACCTGCGACACAAACGG-3′

## Supplementary Materials

The following are available online at https://www.mdpi.com/2072-6643/11/5/1036/s1, Figure S1: Metabolic parameters in HFD-fed and HMCA-supplemented HFD-fed mice, Figure S2: Plasma pharmacokinetic profiles of HMCA and HMPA after intraperitoneal injection in conventional mice, Figure S3: Metabolic parameters in HFD control and HMPA-supplemented HFD-fed mice. Table S1: Daily calorie intake in HMCA consumption, Table S2: Daily calorie intake in HMPA consumption.
