# Supplementary material for: 3-(4-Hydroxy-3-methoxyphenyl)propionic Acid Produced from 4-Hydroxy-3-methoxycinnamic Acid by Gut Microbiota Improves Host Metabolic Condition in Diet-Induced Obese Mice
Source: Nutrients. 2019 May 9;11(5):1036. doi: 10.3390/nu11051036 (PMC6566268; doi:10.3390/nu11051036)
Supplement: Supplementary file 1 [file nutrients-11-01036-s001.pdf]

Supplementary Materials

Table S1. Daily calorie intake in HMCA consumption

	HFD	HMCA
Total calorie intake (kcal/day)	13.75 ± 0.53	12.52 ± 1.11

Table S2. Daily calorie intake in HMPA consumption

	Control	HMPA
Total calorie intake (kcal/day)	11.32 ± 027.	11.78 ± 0.77

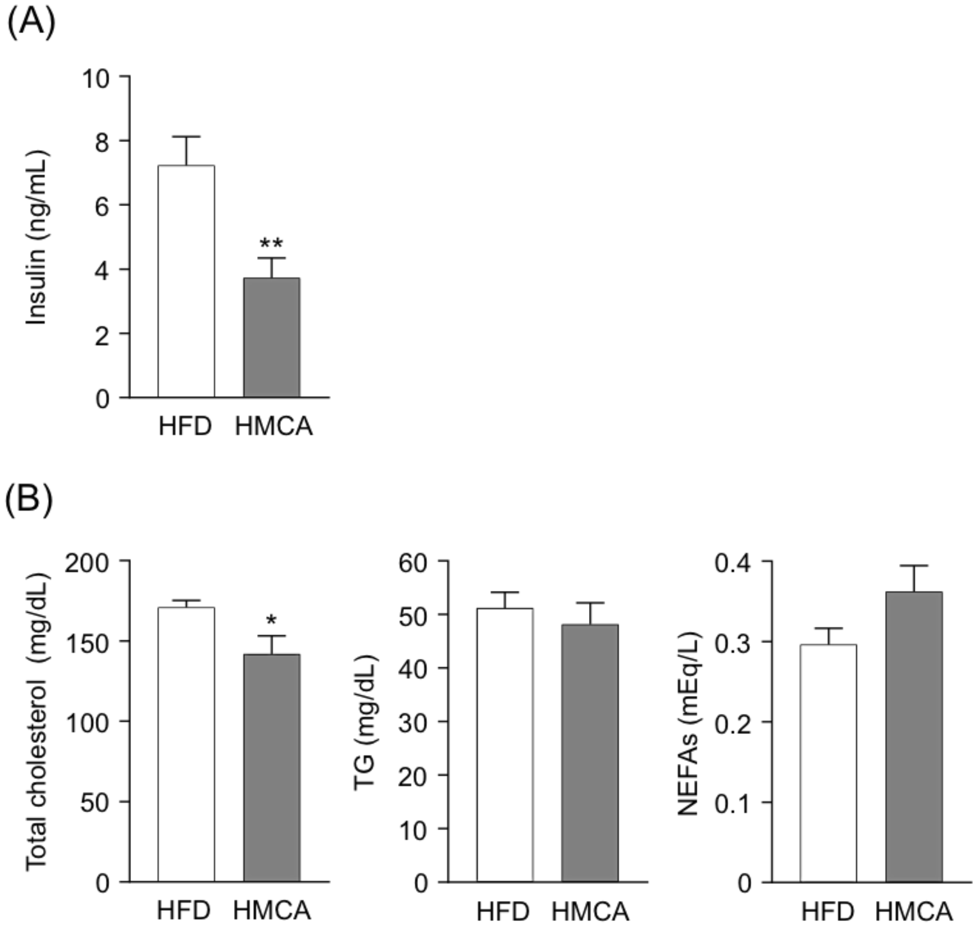

Figure S1. Metabolic parameters in HFD-fed and HMCA-supplemented HFD-fed mice. (A) Plasma insulin was analyzed by ELISA (n = 7–8). (B) Plasma total cholesterol, TG, NEFAs (n = 7–8). All data are presented as the means ± SEM. Differences were assessed by Student's t-test. Significance is established at adjusted **$P < 0.01$ and *$P < 0.05$.

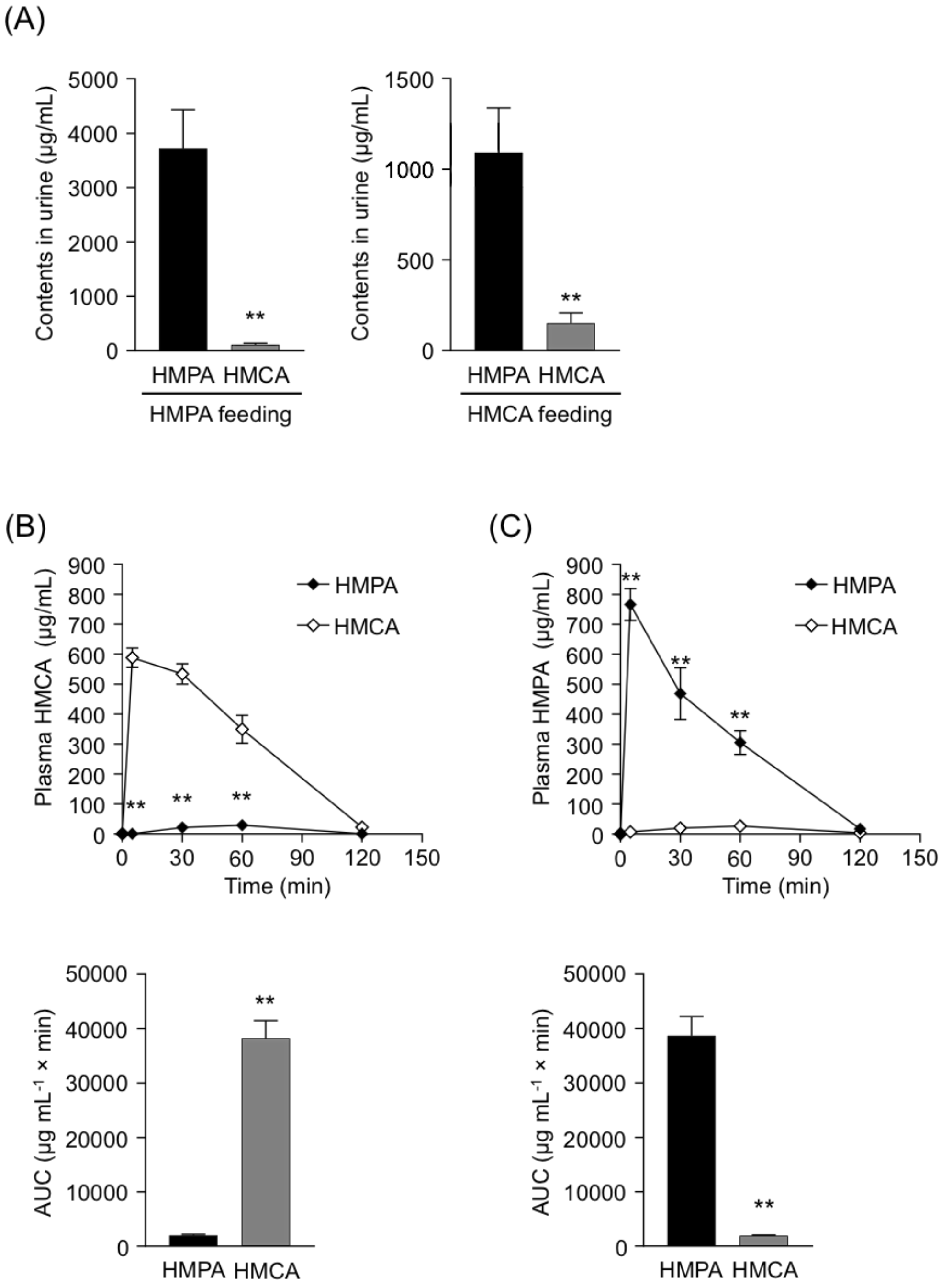

Figure S2. The absorption profile of phytochemicals in HFD-fed mice. (A) Mice were fed HFD containing HMCA or HMPA for one week. After feeding, the urinary contents of HMCA and HMPA were determined (n = 6–8). (B–C) Plasma pharmacokinetic profiles of HMCA and HMPA after intraperitoneal injection in conventional mice. The profile of the HMCA and HMPA detected by HPLC,

according to their time of appearance in plasma following HMCA injection (B) or HMPA injection (C) at 500 mg/kg of body weight, and these area under the curve (AUC), respectively ($n = 3–4$). All data are presented as the means ± SEM. Differences were assessed by Student's t-test. Significance is established at adjusted $**P < 0.01$.

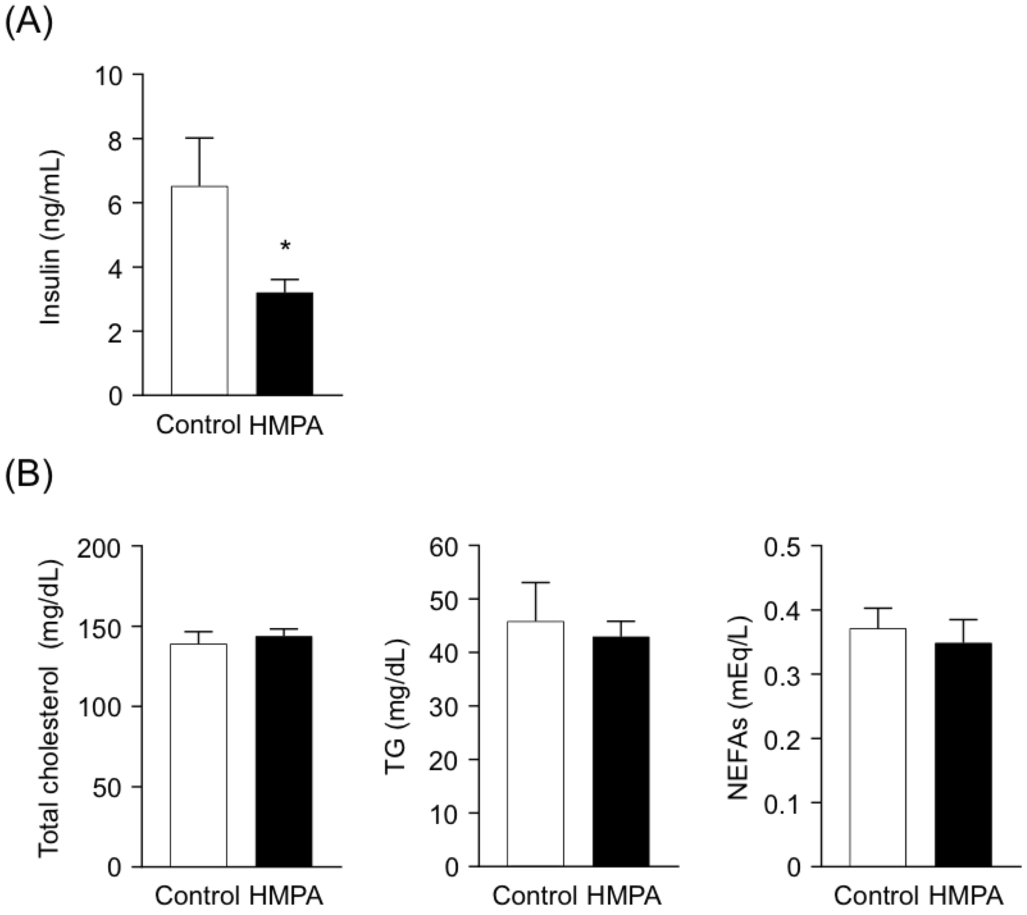

Figure S3. Metabolic parameters in HFD control and HMPA-supplemented HFD-fed mice. (A) Plasma insulin was analyzed by ELISA (n = 7–9). (B) Plasma total cholesterol, TG, NEFAs (n = 7–9). All data are presented as the means ± SEM. Differences were assessed by Student's t-test. Significance is established at adjusted $*P < 0.05$.
